# HDAC2 and 7 down-regulation induces senescence in dermal fibroblasts

**DOI:** 10.18632/aging.203304

**Published:** 2021-07-12

**Authors:** Céline Warnon, Karim Bouhjar, Noëlle Ninane, Mathilde Verhoyen, Antoine Fattaccioli, Maude Fransolet, Catherine Lambert de Rouvroit, Yves Poumay, Géraldine Piel, Denis Mottet, Florence Debacq-Chainiaux

**Affiliations:** 1 URBC, Namur Research Institute for Life Sciences (NARILIS), University of Namur, Namur, Belgium; 2 URPHYM, Namur Research Institute for Life Sciences (NARILIS), University of Namur, Namur, Belgium; 3 Laboratory of Pharmaceutical Technology and Biopharmacy, CIRM, University of Liège, Liège, Belgium; 4 University of Liège, GIGA-Molecular Biology of Diseases, Gene Expression and Cancer Laboratory, Liège, Belgium

**Keywords:** histone deacetylases, senescence, fibroblasts, SAHA, SASP

## Abstract

Originally simply reported to be in a stable and irreversible growth arrest *in vitro*, senescent cells are now clearly associated with normal and pathological ageing *in vivo*. They are characterized by several biomarkers and changes in gene expression that may depend on epigenetic factors, such as histone acetylation, involving a balance between histone acetyltransferases (HATs) and histone deacetylases (HDACs). In this study, we investigate the expression and the role of HDACs on the senescent phenotype of dermal fibroblasts. We report that during replicative senescence, most canonical HDACs are less expressed. Moreover, treatment with SAHA, a histone deacetylase inhibitor (HDACi) also known as Vorinostat, or the specific downregulation of HDAC2 or HDAC7 by siRNA, induces the appearance of senescence biomarkers of dermal fibroblasts. Conversely, the ectopic re-expression of HDAC7 by lentiviral transduction in pre-senescent dermal fibroblasts extends their proliferative lifespan. These results demonstrate that HDACs expression can modulate the senescent phenotype, highlighting their pharmaceutical interest in the context of healthy ageing.

## INTRODUCTION

Ageing is characterized by a general slowdown of the physiological functions of the organism, predisposing to the appearance of age-related pathologies such as cancer, cardiovascular diseases and neurological disorders. Senescent cells, first detected *in vitro*, are now clearly associated with normal and pathological ageing *in vivo* [1, 2]. This has been highlighted thanks to the identification of senescent cells monitored by several biomarkers including the irreversible growth arrest related to the expression of cyclin-dependent kinase inhibitors p16^INK4A^ and p21^WAF-1^, a significant increase in cell size, senescence-associated β-galactosidase activity (SA-βgal), persistent DNA damage, senescence-associated heterochromatin foci (SAHF), resistance to apoptosis, and finally the expression of a particular secretome composed of inflammatory cytokines, chemokines, growth factors and proteases and referred to as the senescence-associated secretory phenotype (SASP) [3, 4]. Senescent cells are suspected to interact with their cellular and matrix microenvironment through this SASP. Expression of SASP factors could be beneficial to the organism by stimulating immune clearance of senescent cells, or by contributing to wound healing through limited fibrosis and activated myofibroblasts differentiation [5]. Nevertheless, some detrimental effects are also associated to the SASP such as the promotion of proliferation, migration and invasion of cancer cells observed *in vitro*, which could possibly contribute to tumoral development *in vivo* [6]. The SASP composition is dependent on the cell type, the senescence inducer and the time spent since the senescence-inducing event [7, 8].

The SASP is mainly regulated at the transcriptional level by several signalling pathways converging to the activation of NF-κB usually in response to DNA double-strands breaks (DSBs) [9]. In senescent cells, permanent and irreparable DSBs create DNA-SCARS (DNA segments with chromatin alterations reinforcing senescence) that generate continuous activation of the DNA Damage Response (DDR) pathway [10, 11]. In addition to the DDR pathway, other signaling pathways can also activate and/or reinforce the SASP, such as p38^MAPK^, JAK/STAT, inflammasome and autophagy [11]. Beside genes coding for SASP factors, other genes also see their expression modified during senescence such as genes encoding proteins involved in the cell cycle. Again, the transcriptomic signature associated with senescence varies depending on the cell type and the inducer of senescence [12].

Gene expression can be regulated at different levels, including epigenetic regulation by which the accessibility of DNA is controlled via modifications of the chromatin structure. Histone post-translational modification such as acetylation can open the chromatin, allowing subsequently the binding of transcription factors which in turn enables gene expression. Acetylation level depends on the balance between the activities of histone acetyltransferases (HATs) and histone deacetylases (HDACs). Mammalian cells express 18 HDAC enzymes that are categorized into two distinct groups: the NAD+-dependent class III HDACs or sirtuins (sirtuin 1-7) and the canonical zinc-dependent HDACs encompassing: class I (HDAC1, 2, 3 and 8), class IIa (HDAC4, 5, 7 and 9), class IIb (HDAC6 and 10) and class IV (HDAC11). Interestingly, HDACs activity diminishes with replicative senescence, leading to an increased histone acetylation [13]. Treatment of normal or cancer cells with HDACi including sodium butyrate (NaB) or trichostatin A (TSA) induces senescence by displaying mainly a p21^WAF-1^-dependent proliferation arrest [14–16]. Moreover, it has been reported that normal immortalized human fibroblasts treated with NaB expressed a SASP via the activation of a non-canonical DDR pathway independently of the ATM kinase activity. This data highlights the fact that epigenetic changes associated to chromatin remodeling are involved in the establishment of the senescent phenotype including the SASP [11, 15]. However, little is known about the changes in HDACs expression during senescence of normal cells and about the contribution of individual HDACs in the onset of senescence. Previous studies have reported that two HDACs were downregulated during replicative senescence of normal cells: HDAC1 in WI-38 fibroblasts [13] and HDAC4 in 2BS fibroblasts [17].

In our study, we demonstrate that expression of most canonical HDACs drastically decreases during replicative senescence of human dermal fibroblasts (HDFs). Treatment of HDFs with SAHA, also known as Vorinostat, a pan-HDACs inhibitor, leads to the establishment of senescence. Moreover, the specific invalidation of HDAC2 and HDAC7 gene expression by siRNA also induces a premature appearance of the senescent phenotype. Conversely, the ectopic re-expression of HDAC7 by lentiviral transduction extends the proliferative lifespan of the pre-senescent HDFs. These results demonstrate that a global repression of canonical HDACs is detected during replicative senescence of normal HDFs and that the modulation of the expression of some of them individually can impact the senescent phenotype. This reinforces their interest as potential pharmaceutical targets in the context of healthy ageing.

## RESULTS

### HDACs expression is decreased in dermal HDFs during replicative senescence

**Figure 1:**
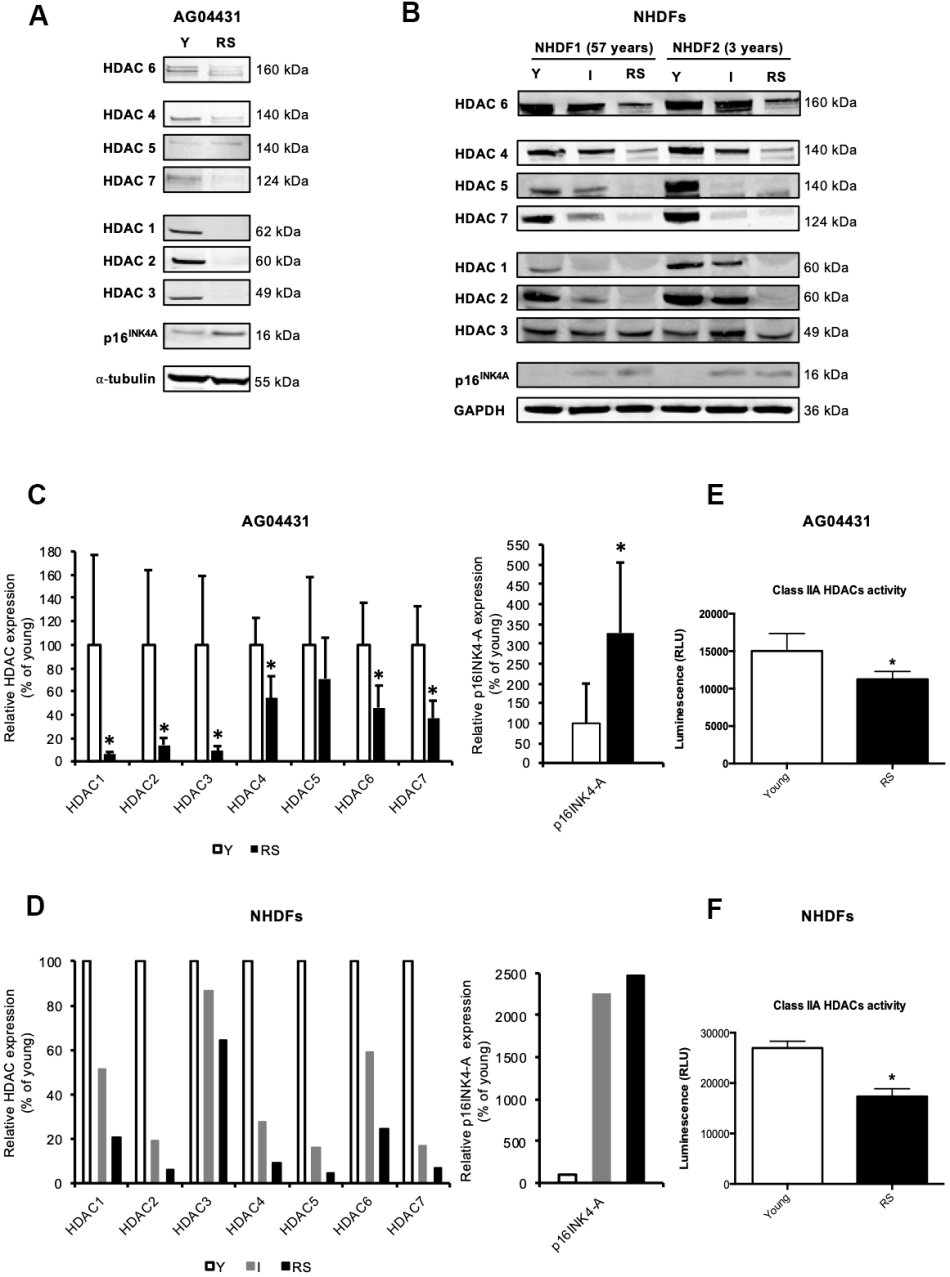
**Reduced expression of HDACs expression in replicative senescence.** (**A**, **B**) Representative Western blots showing HDACs 1-7 and p16^INK-4A^ protein level in young (early passage, Y), intermediate (I) or replicative senescent (RS) AG04431 cells (**A**) or primary normal human dermal fibroblasts (NHDFs) isolated from an adult donor (57 years) or a young donor (3 years) (**B**). (**C**, **D**) Quantifications of the protein level of HDACs 1-7 and p16 ^INK-4A^, with α-tubulin (AG04431, n=4) or GAPDH (NHDFs, n=2) as loading control. (**E**, **F**) Class IIa HDACs activity in AG04431 (e) (n=3) or in NHDFs (**F**) (n=3). Statistical analyses were performed using a *t*-test (*: p<0.05).

Except for a decreased expression of HDAC1 [13] and HDAC4 [17] during replicative senescence of fibroblasts, changes in the expression of other canonical HDACs during senescence have not been reported. To investigate whether the expression of other HDACs is also modified during replicative senescence of human dermal fibroblasts (HDFs), we monitored the protein levels of HDACs 1-7 in three HDFs strains: AG04431 human dermal fibroblasts and normal HDFs isolated from 57-yrs-old (NHDF1) and 3-yrs-old (NHDF2) donors. Dermal fibroblasts were passaged in cell culture until replicative senescence (RS), which occured namely when cells reached stable growth-arrest for at least two weeks and when more than 70% of the cell population was SA-βgal positive. HDACs 1-7 proteins levels displayed a general decrease in RS as compared to fibroblasts at early passage (young) (Figure 1A, 1B). This decrease in protein abundance could vary between 29% (HDAC5) and 98% (HDAC2) (Figure 1C, 1D), and seemed progressive as shown in an intermediate passage for NHDFs, corresponding to the middle of their proliferative lifespan (Figure 1B, 1D and Supplementary Figure 1). As a control, we confirmed an increase in the protein abundance of p16^INK4A^ in RS conditions. The overall decrease in protein abundance of HDACs is corroborated with a significant decrease in the levels of transcripts encoding *HDAC1-3*, *6*, and *7* in RS, compared to early passage (young) AG04431 (Supplementary Figure 2). In order to study whether this difference in protein expression was associated with a difference in enzymatic activity, we carried out a class IIa HDACs activity assay. We observed a decrease in class IIa HDACs activity between young and RS HDFs (Figure 1E, 1F). Together, these results suggest a global decrease in HDACs expression and activity during replicative senescence of dermal fibroblasts.

### SAHA treatment in young HDFs promotes the appearance of senescence-associated biomarkers

**Figure 2:**
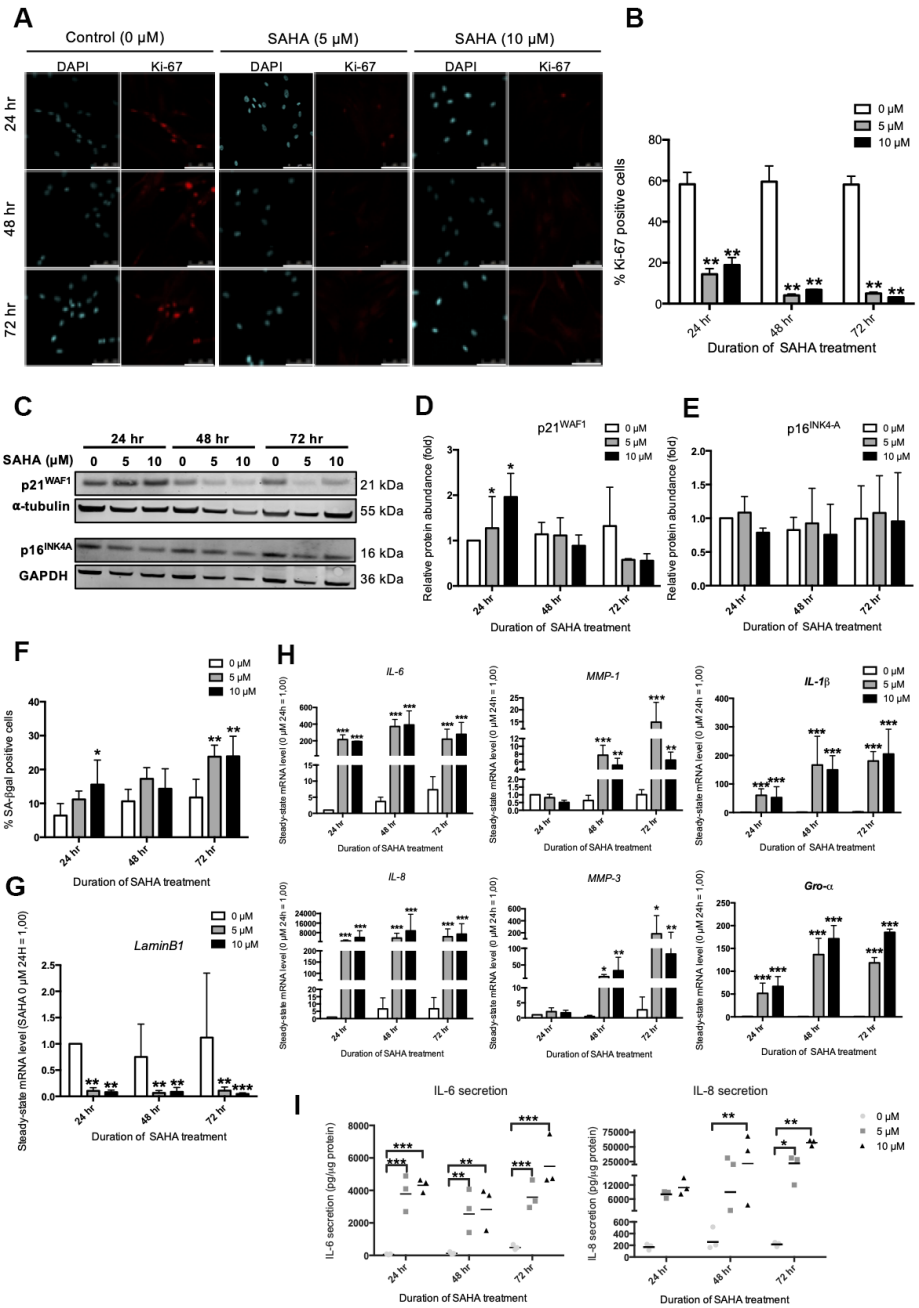
**SAHA repeated treatment induces the appearance of senescence biomarkers in AG04431 cells.** Cells at early passage were treated with 0, 5 or 10 μM of SAHA during 24, 48 or 72 hr. (**A**) Representative confocal images of cells labelled with Ki-67 staining (red) and DAPI (nucleus staining, blue) (scale bar = 50 μM). (**B**) Percentage of Ki-67-positive cells. (**C**) Representative Western blots showing p16^INK-4a^ and p21^WAF-1^ protein level, with α-tubulin or GAPDH as loading control. (**D**, **E**) Quantifications of the relative protein level of p21^WAF-1^ (**D**) and p16^INK-4a^ (**E**). Signal intensities were quantified and normalized according to the abundance of α-tubulin or GAPDH and were expressed relatively to the control condition (0 μM SAHA, 24 hr). (**F**) Percentage of SA-βgal positive cells. (**G**) Steady-state mRNA level of *Lamin B1*. *GAPDH* was used as a housekeeping gene. (**H**) Steady-state mRNA level of *IL-6*, *IL-8, MMP-1, MMP-3, IL-1β* and *Gro-α*. *GAPDH* was used as housekeeping gene. Results are normalized to the control condition (0 μM SAHA, 24 hr). (**I**) Secretion of IL-6 and IL-8 following SAHA treatment. Supernatants were collected at 24, 48 and 72 hr and the IL-6 and IL-8 secreted levels were monitored by ELISA. Statistical analyses were performed using an ANOVA II (*: p<0.05; **: p<0.01; ***: p<0.001).

In order to mimic the impact of this overall decrease in HDACs expression during RS, the inhibition of HDACs was studied using SAHA, a pan-HDACs inhibitor. In cancer cell lines and in normal fibroblasts, SAHA was shown to induce several senescence biomarkers such as growth arrest and an increased proportion of SA-βgal positive cells [18, 19]. Dermal fibroblasts were incubated with SAHA during 24 hr, 48 hr and 72 hr and several biomarkers of senescence were assessed, including the expression of several SASP factors. Subcytotoxic doses of 5 and 10 μM of SAHA were used (Supplementary Figure 3A, 3B). A dose-dependent increase in the acetylation of Histone H3 was observed following SAHA treatment (Supplementary Figure 4A, 4B), as well as a decreased activity of class IIa HDACs (Supplementary Figure 4C). The impact of SAHA treatment on the senescent phenotype of fibroblasts was then analysed. The effect of SAHA on proliferation was determined by the proportion of Ki-67 positive cells [20]. A strong decrease in the proportion of Ki-67 positive cells is observed after SAHA treatment regardless of the dose and duration of the treatment (Figure 2A, 2B). The protein level of p16^INK4-A^ and p21^WAF-1^, two CDKIs that are associated with growth-arrest in senescent cells, were then assessed. The expression of p21^WAF1^ protein was increased after 24 hours of treatment with both doses (Figure 2C, 2D), while the protein level of p16^INK4-A^ remained stable (Figure 2C, 2E). This result is consistent with previous studies that have described increased expression of p21^WAF1^ after SAHA treatment [21]. In addition, the proportion of SA-βgal positive cells significantly increased in response to SAHA treatment after 72 hours (Figure 2F). The effect of SAHA on *Lamin B1 (LMNB1)* mRNA levels was then analysed and the results demonstrated a strong decrease of *LMNB1* transcript levels following treatment (Figure 2G). Lamin B1 is a protein of the *nuclear lamina*, lining the inner surface of the nuclear envelope, and is now considered as a robust biomarker of senescence as its expression is decreased in several models of senescence [22]. Regarding the effect of SAHA treatment on SASP factors expression, SAHA treatment induced an increase in the expression of *IL-6, IL-8 (CXCL8), MMP-1*, *MMP-3*, *IL-1β* and *Gro-α (CXCL1)* at the mRNA level, whatever the dose and the duration of treatment (Figure 2H). Moreover, the secretion of IL-6 and IL-8 significantly increased from 24 hours for IL-6, and from 48 hours for IL-8 (Figure 2I), confirming a rapid effect of SAHA on SASP induction, as recently reported following the treatment of HDFs with sodium butyrate (NaB) and trichostatin A (TSA), two other HDACi [15].

Altogether, these results demonstrate that the treatment of young dermal HDFs with SAHA induces the premature onset of senescence in dermal fibroblasts, including the expression of some SASP factors.

### siRNA targeted inhibition of HDAC2 and HDAC7 induces senescent-like phenotype in young HDFs

**Figure 3:**
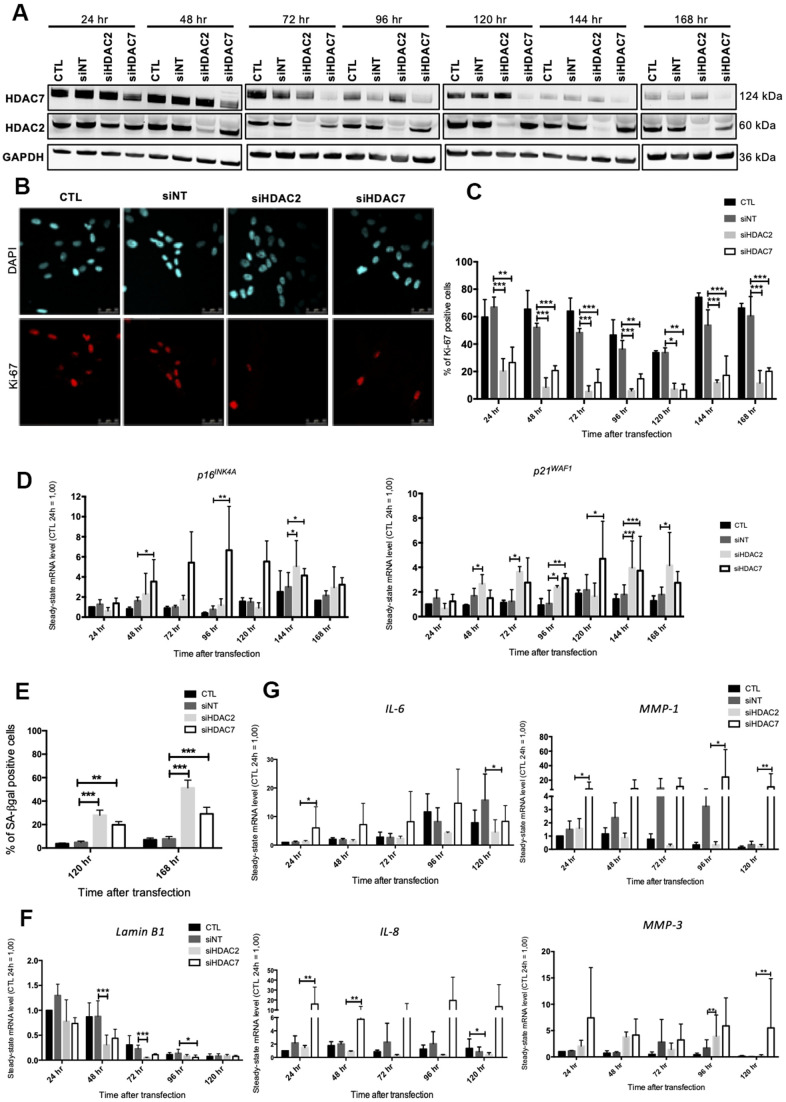
**Knockdown of HDAC2 or HDAC7 induces senescence in AG04431 cells.** Cells at early passage were transfected with control siRNA (non target, siNT), HDAC2 siRNA (siHDAC2) or HDAC7 siRNA (siHDAC7) during 24 hr and biomarkers of senescence were analysed every day during 7 days. (**A**) Representative Western blots showing HDAC2 and HDAC7 total protein abundance at different times (24-168 hr) after siRNA transfection. GAPDH was used as loading control. (**B**) Representative confocal images of cells labelled with Ki-67 staining (red) and DAPI (nucleus staining, blue) (scale bar = 50 μM). (**C**) Percentage of Ki-67-positive cells. (**D**) Steady-state mRNA level of *p16^INK-4a^* and *p21^WAF-1^*. *GAPDH* was used as a housekeeping gene. Results are expressed as fold induction in comparison to control fibroblasts at 24 hr. (**E**) Percentage of SA-βgal positive cells. (**F**) Steady-state mRNA level of *Lamin B1*. *GAPDH* was used as a housekeeping gene. (**G**) Steady-state mRNA level of *IL-6, IL-8, MMP-1 and MMP-3*. *GAPDH* was used as housekeeping gene. Results are expressed as fold induction in comparison to control fibroblasts at 24 hr. Statistical analyses were performed using an ANOVA II (*: p<0.05; **: p<0.01; ***: p<0.001).

As SAHA is a pan HDACi, it is not possible to know if its effects are associated with the inhibition of one or more HDACs. In order to decipher whether the premature senescence phenotype associated to HDACs inhibition is related to a specific HDAC, we performed a targeted invalidation by siRNA of two HDACs of different classes whose expression levels were reported to be highly diminished in senescent fibroblasts, *i.e*. HDAC2 and HDAC7. The invalidation of *HDAC2* and *HDAC7* has been reported to be associated with proliferation arrest respectively in MCF-7 breast cancer cell line [23] and in mucoepidermoid carcinoma cells [24], but not yet investigated on the potential induction of the senescent phenotype in normal cells. Dermal HDFs were transfected with siRNA targeting either *HDAC2* or *HDAC7* during 24 hr and maintained in culture for seven days. *HDAC2* and *HDAC7* transcript levels were decreased from 24 hr post-transfection to at least 168 hr post-transfection (Supplementary Figure 5). Regarding the protein level, HDAC2 and HDAC7 were decreased from 48 hr after siRNA transfection and remained low until 168 hr (Figure 3A). No impact of the invalidation of *HDAC2* on the protein abundance of HDAC7 was observed, and vice versa (Figure 3A). This observation confirmed the proper inhibition of HDAC2 and HDAC7 expression by using specific siRNA.

The impact of *HDAC2* or *HDAC7* invalidation on proliferation was then tested. Results demonstrated that *HDAC2* and *HDAC7* invalidation strongly reduced the proportion of Ki-67 positive cells, starting 24 hr after transfection (Figure 3B, 3C). This decrease in proliferation was correlated to an increase in the mRNA level of *p16^INK4-A^* and *p21^WAF-1^* from 48 hr to 168 hr post-transfection (Figure 3D). At the protein level, results displayed a significant increase in the protein abundance of p16^INK4-A^ at 120 hr post-transfection for siHDAC7, as well as a tendency for an increase in protein expression of p21^WAF-1^, for both HDACs siRNA, at 144 hr (Supplementary Figure 6). The proportion of SA-βgal positive cells following *HDAC2* and *HDAC7* invalidation showed a significant increase in both siHDAC2 and siHDAC7 transfected cells, compared to cells transfected with the non-targeting siRNA (siNT) at 120 hr and 168 hr post transfection (Figure 3E). The transcript levels of *LMNB1* was then studied and results demonstrated a decrease in *LMNB1* transcript levels at 48 hr and 72 hr in HDFs transfected with siHDAC2 and at 96 hr in HDFs transfected with siHDAC7 (Figure 3F). The effect of siHDAC2 or siHDAC7 transfection on the expression of several SASP factors displayed different results between the two siRNAs. HDFs transfected with siHDAC7 overexpressed significantly *IL-6*, *IL-8*, *MMP-1* and *MMP-3* at some time points (Figure 3G). Conversely, transfection of HDFs with siHDAC2 showed minor effects on the expression of these SASP factors, as only a significant increase in the expression of *MMP-3* was detected at 96 hr post-transfection (Figure 3G).

These results demonstrate that the transfection of young HDFs with siHDAC2 or siHDAC7 induces a senescent phenotype highlighted by a growth-arrest, an increase in the proportion of SA-βgal positive cells and a decreased expression of *LMNB1*. However, only siHDAC7 has an impact on the expression levels of several SASP factors, and is subsequently able to reproduce a senescent phenotype similar to the one obtained upon SAHA treatment.

### Re-expression of HDAC7 delays the occurrence of the cell cycle arrest in pre-senescent dermal fibroblasts

**Figure 4:**
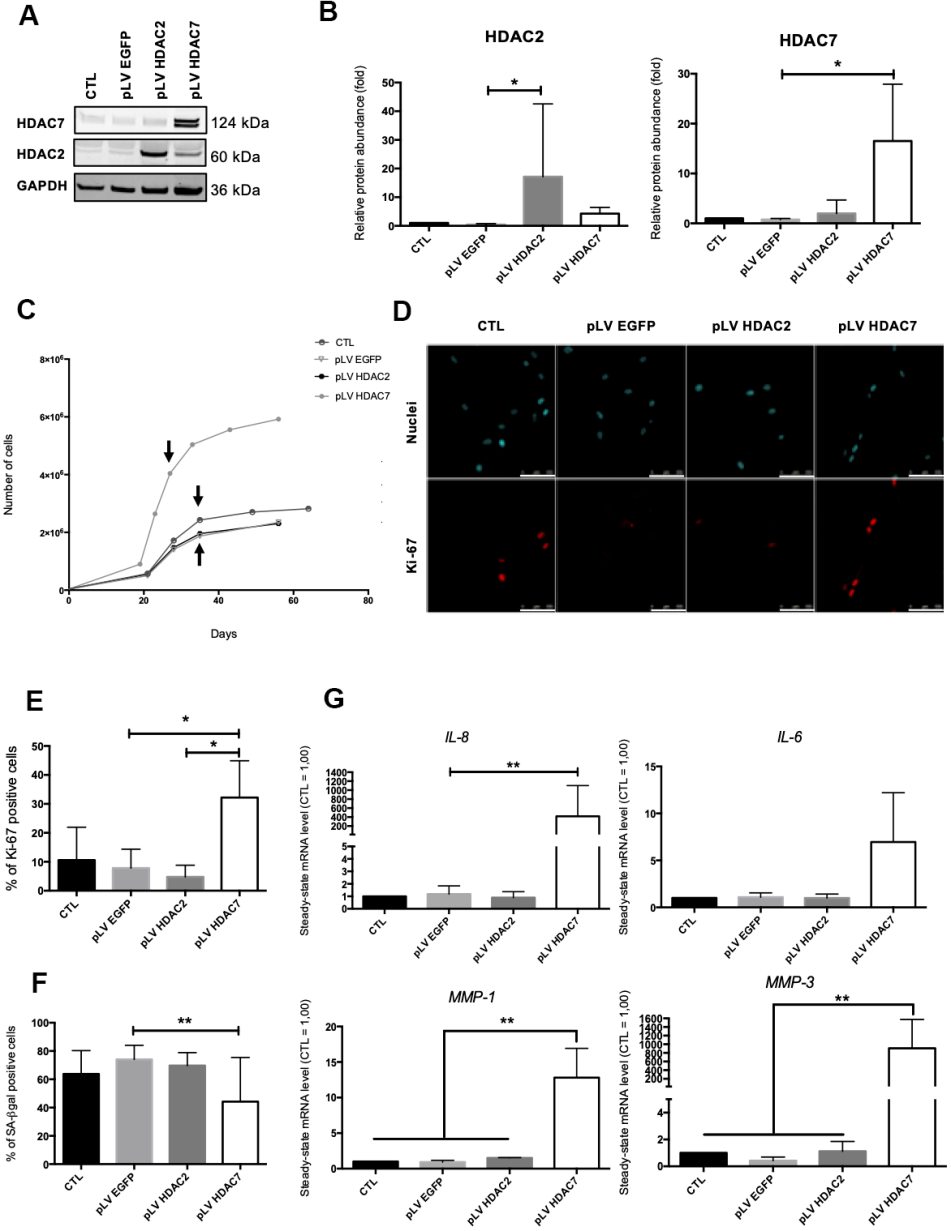
**HDAC7 but not HDAC2 re-expression allows to resume proliferation in pre-senescent cells.** Pre-senescent AG04431 HDFs, *i.e.* cells at few passages from the onset of replicative senescence, were transduced with lentiviruses expressing EGFP (pLV EGFP), HDAC2 (pLV HDAC2) or HDAC7 (pLV HDAC7). (**A**) Representative Western blots showing HDAC2 and HDAC7 expression after transduction. GAPDH was used as a loading control. (**B**) Quantification of the relative protein abundance of HDAC2 and HDAC7. Signal intensities were quantified and normalized relative to the abundance of GAPDH and are expressed relatively to the control condition (CTL). (**C**) Representative growth curves of the cells with indicated conditions. The passages studied are indicated by an arrow. (**D**) Representative confocal images of cells labelled with Ki-67 staining (red) and DAPI (nucleus staining, blue) (scale bar = 50 μM). (**E**) Percentage of Ki-67-positive cells. (**F**) Percentage of SA-βgal positive cells. (**G**) Steady-state mRNA level of *IL-6, IL-8*, *MMP-1* and *MMP-3*. *GAPDH* was used as housekeeping gene. Results were expressed as fold induction in comparison with the control condition. Statistical analyses were performed using an ANOVA I (*: p<0.05; **: p<0.01).

Previous results have shown that the inhibition of HDACs with the use of SAHA or siRNA induced the premature appearance of senescence in dermal fibroblasts. The analysis of the re-expression of *HDAC2* or *HDAC7* expression in pre-senescent cells, namely cells at a few passages to enter in replicative senescence, but still able to proliferate was then addressed. For this purpose, we infected AG04431 HDFs and dermal NHDFs with lentivirus expressing HDAC2 (pLV HDAC2), HDAC7 (pLV HDAC7), or enhanced green fluorescent protein (pLV EGFP) as a control. The increase in the protein levels of HDAC2 or HDAC7 in transduced pre-senescent cells was monitored by Western blot (Figure 4A, 4B and Supplementary Figure 7A, 7B). After transduction, cells were passaged until they reached replicative senescence. Analyses were carried out at passage 4 post transduction for all replicates and for both AG04431 HDFs and NHDFs (corresponding to day 28 for pLV HDAC7 and to day 36 for CTL, pLV EGFP and pLV HDAC2). We first analysed the effect of transduction on cell proliferation. Both HDFs and NHDFs cells transduced with pLV HDAC7 exhibited a resumption of their proliferation as well as an extension of their proliferative lifespan, as shown by an increased cell number (Figure 4C and Supplementary Figure 7C). Moreover, cells transduced with pLV HDAC7displayed an increased proportion of Ki-67 positive signals (Figure 4D, 4E). Re-expression of HDAC7 also results in a significant decrease in the proportion of SA-βgal positive cells (Figure 4F and Supplementary Figure 7D). Moreover, representative cell images demonstrated that HDFs transduced with pLV HDAC7 displayed a more spindle-shaped morphology, resembling young proliferative cells (Supplementary Figure 8). The effect of transduction on the expression of SASP factors was then assessed. Surprisingly, an increased expression of *IL-6, IL-8, MMP-1* and *MMP-3* was observed in the HDFs transduced with pLV HDAC7 compared with other conditions (Figure 4G). *HDAC7* re-expression leads to the extension of the proliferative capacities of HDFs as well as to a decrease in the proportion of SA-βgal positive cells. However, it is not associated with a reduced expression of SASP factors.

### NF-ĸB activation is partly involved in IL-6 and IL-8 expression after HDACs inhibition in young HDFs

Our results pointed out that HDACs inhibition in AG04431 dermal fibroblasts, either with SAHA or with siRNA targeting *HDAC2* or *HDAC7* induced a strong overexpression of some SASP factors, such as *IL-6*, *IL-8*, *MMP-1* and *MMP-3.* SASP regulation and particularly *IL-6* and *IL-8* expression*,* is largely dependent on NF-κB (nuclear factor-kappa B) activation [11].

**Figure 5:**
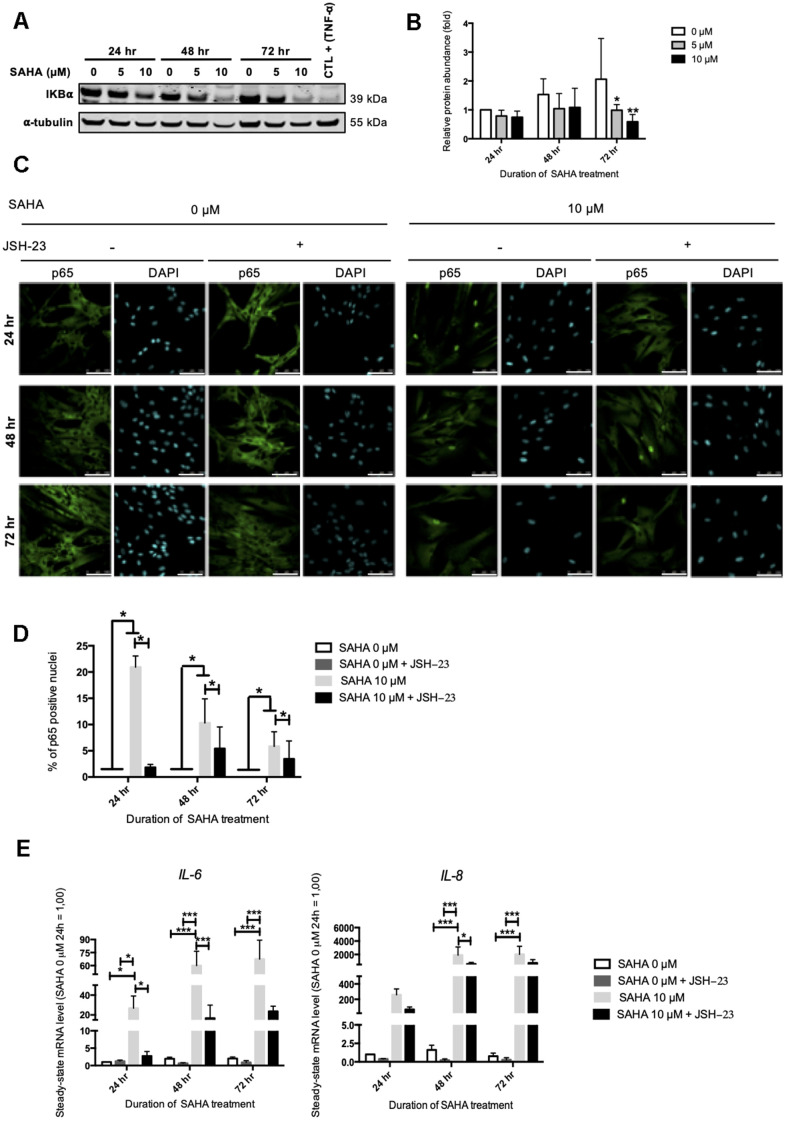
**IL-6 and IL-8 expression observed after SAHA treatment is partly dependent on NF-κB activation.** Cells at early passage were treated with 0, 5 or 10 μM of SAHA during 24, 48 or 72 hr in combination or not with JSH-23 treatment (NF-κB inhibitor) during 24 hr. (**A**) Representative Western blots showing IĸBα total protein abundance after SAHA treatment. AG04431 dermal fibroblasts treated with 20 ng/mL of TNF-α during 20 minutes were used as positive control for IĸBα degradation. α-tubulin was used as loading control. (**B**) Quantification of the relative protein abundance of IĸBα. Signal intensities were quantified and normalized relative to the abundance of α-tubulin and are expressed relative to the control condition (0 μM SAHA, 24 hr). (**C**) Immunofluorescence analysis of p65 (green) nuclear translocation. Nuclei were labelled with DAPI (blue). Cells were visualized with confocal microscopy (scale bar = 50 μM). (**D**) Quantification of the percentage of p65 positive nuclei. (**E**) Steady-state mRNA level of *IL-6* and *IL-8*. *GAPDH* was used as housekeeping gene. Results are expressed as fold induction in comparison with control fibroblasts (0 μM SAHA, 24 hr). Statistical analyses were performed using an ANOVA II (*: p<0.05; **: p<0.01; ***: p<0.001).

NF-κB is a transcription factor composed of several subunits that is sequestered in the cytoplasm by IκBα (NF-κB inhibitor alpha), keeping it in an inactive state and preventing its nuclear translocation. In the presence of an activating signal, the activation of IκBs kinases (IKKs) leads to the phosphorylation and the proteasomal degradation of IκBα, allowing the nuclear translocation of NF-κB and the activation of target genes. In addition to NF-κB, the expression of *IL-6* and *IL-8* can be dependent on other transcription factors such as C/EBPβ (CCAAT/enhancer binding protein beta) [25]. The protein level of IκBα after SAHA treatment was first assessed. SAHA treatment in AG04331 HDFs induced the degradation of IκBα after 72 hr (Figure 5A, 5B). We confirmed the activation of NF-κB as shown by an increase of p65 nuclear translocation upon SAHA treatment (Figure 5C, 5D). In order to decipher whether the levels of expression of SASP factors after SAHA treatment were dependent on NF-κB activation, we performed NF-κB inhibition using JSH-23, an inhibitor of p65 nuclear translocation. The data highlighted that in cells treated with JSH-23 concomitantly with SAHA treatment, the nuclear signal of p65 is strongly reduced, consolidating the nuclear translocation of NF-κB following SAHA treatment (Figure 5C, 5D). The combined treatment of dermal HDFs with JSH-23 and SAHA significantly reduced the expression of *IL-6* and *IL-8* (Figure 5E). These results reinforce the link between NF-κB activation and the expression of SASP factors following SAHA treatment.

The analysis of IκBα protein abundance after *HDAC2* or *HDAC7* siRNA transfection displayed a slight decrease at 96 hr following transfection with HDAC7 siRNA, suggesting a possible NF-κB activation in this condition (Supplementary Figure 9). These results confirm the possible involvement of NF-κB activation in the regulation of the expression of SASP factors upon HDACs inhibition either through treatment using SAHA or through targeted siRNA invalidation.

## DISCUSSION

Several studies reported a link between HDACs inhibition, growth arrest and SA-βgal activity in a broad range of cancer cell lines. Therefore, HDACi are used in anticancer therapies, allowing the induction of senescence in cancer cells while having low toxicity in normal cells [26]. This particular type of senescence is called therapy-induced senescence (TIS) and is widely used for cancer treatments [27]. Indeed, the synergistic combination of HDACi and classical chemotherapy has demonstrated very encouraging results to face cancer, such as cisplatin or etoposide in combination with valproic acid in neuroblastoma cells [28] or with belinostat or romidepsin in small cell lung cancer (SCLC) [29], or even temozolomide (TMZ) in combination with valproic acid in glioma cells [30]. However, studies dealing with the role of HDACs in the onset of senescence in normal cells remain scarce. HDAC1 and HDAC4 have been reported to be downregulated in replicative senescent normal fibroblasts [13, 17]. Moreover, the use of HDACi has been shown to induce senescence biomarkers, such as SA-βgal, growth-arrest [13] and expression of several SASP factors [15, 31].

In this study, we present strong evidence supporting the major role played by HDAC2 and HDAC7 in the priming of the senescent phenotype of dermal fibroblasts. Indeed, we first analysed the global expression of HDACs during replicative senescence in dermal fibroblasts of different origins. Results herein demonstrate that HDACs 1-7 protein abundance decreases during replicative senescence. Simultaneously, the activity of class IIa HDACs is diminished, suggesting that the decrease in HDACs protein abundance directly impacts their overall activity. Previous data have shown a link between cell cycle-arrest and HDACs expression. This link may be explained by interactions of HDACs with retinoblastoma protein (RB) [32]. In fact, RB binds to the activation domain of E_2_F, causing its inactivation, and is able to interact with HDACs acting as co-repressors. This association is responsible for histone deacetylation in promoters which control expression of cell cycle regulatory genes, such as *cyclin A*, *cdc2*, *topoisomerase IIα* or *thymidylate synthase*, an event that results in transcriptional repression of those genes [33]. However, the presence of HDAC activity seems to be dispensable for the maintenance of cell cycle arrest, since the inhibition of HDACs with trichostatin A (TSA) does not restore cell proliferation [33]. The effect of HDACs on cell proliferation could also be explained by the acetylation levels of promoters of genes coding for cyclin-dependent kinase inhibitors (CDKIs). A study in hepatocellular carcinoma (HCC) cells has indeed demonstrated that inhibition of HDAC1 and HDAC2 triggered increased expression of p21^WAF1^ and p19^INK4D^ and subsequent growth arrest. The promoters of these genes are inactive when they are hypoacetylated and HDACs inhibition allows their acetylation, and consequently their transcriptional activity [34]. Interestingly, this effect on p21^WAF1^ promoter acetylation has been confirmed in cancer cells but not in normal cells [35], suggesting a different regulation. HDACs 1 and 2 interact and regulate promoter activation of p16^INK4A^ in 2BS cells [36]. The inhibition of p16^INK4A^ promoter by HDAC3 and HDAC4 has been detected in NCI-H460 lung cancer cells [37]. Finally, HDACs exhibit a silencing effect on promoters for other cell cycle regulators, such as p15^INK4B^ [38]. Regarding our results, the decrease in HDACs expression and activity during replicative senescence could be part of the onset of senescence. Indeed, the decreased activity of HDACs could enhance expression of cell cycle regulators, such as p16^INK4-A^ or p21^WAF1^. Since we demonstrated that HDACs expression is decreased during replicative senescence, we next investigated the effect of HDACs inhibition on the onset of senescence. The consequences of HDAC inhibition were tested by the use of SAHA, a HDACi that completely inhibits both class I and class II HDACs, and by the use of specific siRNAs targeting *HDAC2* or *HDAC7*. SAHA has been largely used for HDACs inhibition in cancer cells and is approved by the FDA for the treatment of cutaneous T-cell lymphoma. We hereby demonstrate that treatment of young AG04431 HDFs with SAHA induces cell proliferation arrest (as detected by a decreased staining of Ki-67 in these cells), concomitant with an increased protein abundance of p21^WAF-1^, an increase in the proportion of SA-βgal positive cells, a decreased expression of *Lamin B1* and an increased expression and secretion of several SASP factors. These results concur with the induction of biomarkers of senescence, including growth arrest, following SAHA treatment in human leukemia cell lines [39], in rhabdomyosarcoma cell lines [40] and in human colon cancer cell line HCT116 [41]. As p21^WAF-1^ protein abundance is increased at 24 hr after SAHA treatment, it could be involved in the early proliferative arrest. However, at 48 hr and 72 hr, the protein abundance levels of p16^INK4-A^ and p21^WAF-1^ returned to values close to the control, or even lower for p21^WAF-1^ at 72 hr, while the proportion of SA-βgal positive cells in clearly increased. This is consistent with a previous study led by Munro J et al. [42] revealing that the treatment of human fetal fibroblasts with sodium dibutyrate induced a swift overexpression of p16^INK4-A^ and p21^WAF-1^, while the proportion of SA-βgal positive cells was limited, followed by the disappearance of p21^WAF-1^ and an increased proportion of SA-βgal positive cells. Thus p21^WAF-1^ appears to be involved in the immediate cell cycle arrest following treatment with chemical inhibitors of HDACs while the maintenance of proliferation arrest in treated cells could depend on the expression of other cell cycle arrest inhibitors. Previous studies have indeed demonstrated the involvement of p19^INK4D^ in proliferative arrest after HDAC1 and HDAC2 inhibition in hepatocellular carcinoma cells [34], as well as the involvement of p15^INK4B^ after treatment of HaCat cells with TSA or NaB [38]. Moreover, these authors confirm the p21^WAF-1^-independent cell cycle arrest with the use of p21^WAF-1^-deleted human colorectal carcinoma cell line (HCT116 p21^-/-^). These two pathways could therefore also be involved in cell cycle arrest in our conditions. Moreover, it is also possible that the expression of the different CDKIs increases by waves at different timings. This hypothesis fits with results obtained after siRNA knockdown of *HDAC2* and *HDAC7*, with an increased expression of p16^INK4A^ and p21^WAF-1^ at 120 hr and 144 hr post-transfection.

Furthermore, treatment of normal fibroblasts with different HDACi leads to the rapid expression of several SASP factors, by a non-canonical DDR in the absence of DNA damage, but dependent of NF-κB activation [31, 15]. This leads to modifications in chromatin organization that could impact subsequent gene expression [43]. Treatment with HDACi is therefore very informative but does not allow to discriminate which HDAC, impacted by the inhibition, plays a role in the observed phenotype. This is the reason why we completed our study by testing the specific siRNA knockdown of *HDAC2* or *HDAC7*. We selected both HDACs as they belong to two different classes of HDACs and as their expression is strongly decreased during replicative senescence of dermal fibroblasts. Our results demonstrate that *HDAC2* and *HDAC7* invalidation induces the appearance of senescence biomarkers, such as cell cycle arrest, the increased proportion of SA-βgal positive cells, the decreased expression of *Lamin B1* and the overexpression of several SASP factors. To date, this is the first study demonstrating the role of HDAC2 or HDAC7 in the senescent phenotype of a normal cell type. However, the impact of *HDAC2* and *HDAC7* invalidation on the expression of SASP factors differs between both HDACs. Indeed, the knockdown of *HDAC7* leads to increased gene expression of *IL-6*, *IL-8*, *MMP-1* and *MMP-3*, while *HDAC2* invalidation only increases the expression of *MMP-3* at a single timepoint. This is, to our knowledge, the first report linking *HDAC7* specific knockdown to SASP expression, whilst a previous study had correlated *HDAC2* knockdown and overexpression of TNF-α in T cells [44].

In order to investigate whether this decrease in HDAC2 and HDAC7 expression was also involved during replicative senescence, we re-expressed *HDAC2* and *HDAC7* in pre-senescent cells (AG04431 HDFs and NHDFs), a few passages before their entry into replicative senescence. The re-expression of *HDAC7* but not *HDAC2* allows to resume proliferation and to decrease the proportion of SA- βgal positive cells, but this was accompanied by a surprising overexpression of *IL-6*, *IL-8*, *MMP-1* and *MMP-3*. Interestingly, another study pointed out that HDAC4 overexpression in human fibroblasts delays senescence induction via a decreased proportion of SA-βgal positive cells, and an increased proliferative lifespan [17]. The discrepancy we observed in cell response between proliferation markers and SASP expression factors could be due to different kinetics. In fact, it is now widely accepted that SASP is a temporally regulated program [11], some factors being expressed soon after the induction of senescence, while others are expressed only after several days, for long-term senescence. For technical reasons, we could only analyse the expression of the SASP factors after several passages in culture after transduction, which may have diluted any effect.

The pathway inducing SASP-related genes overexpression after HDACs inhibition and invalidation was then investigated. Our results suggest that NF-κB, a master regulator of the expression of SASP factors, is activated after HDACs inhibition, either by SAHA or by targeted invalidation of HDAC7. Indeed, we demonstrated a decreased protein abundance of IκBα, suggestive of NF-κB activation [45]. The activation of NF-κB was also demonstrated by the nuclear translocation of p65 after SAHA treatment. Moreover, our results demonstrate that NF-κB is, at least partly, responsible for the induction of *IL-6* and *IL-8* expression as the use of JSH-23, an inhibitor of the nuclear translocation of p65 [46], prevents their overexpression following SAHA treatment. However, since JSH-23 does not completely abrogate the overexpression of *IL-6* and *IL-8*, it is likely that other pathways are involved, for example the induction of C/EBPβ (CCAAT/enhancer binding protein beta), another transcription factor implicated in the expression of SASP factors [25]. Some studies have previously demonstrated that inhibition of the NF-κB pathway after treatment with HDACs inhibitors, such as NaB, reduces *IL-6* and *IL-8* expression [31]. However, this is the first study showing this mechanism with SAHA or using siRNA targeting *HDAC2* or *HDAC7*.

**Figure 6:**
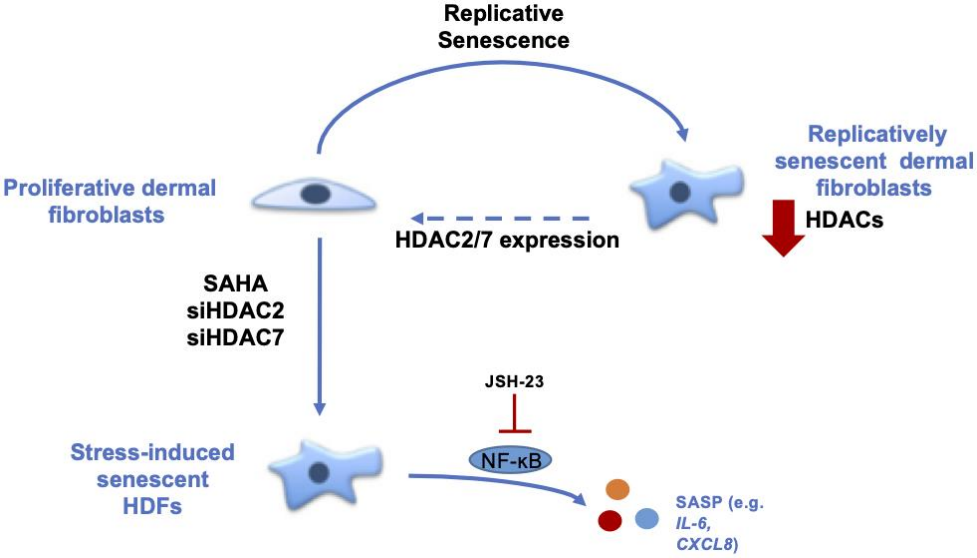
**Summary about the role of HDACs in the senescent phenotype of dermal fibroblasts.** During replicative senescence of skin cells, HDACs protein abundance is reduced. The inhibition of HDACs with the use of SAHA, a pan-HDACs inhibitor, as well as the targeted knockdown of HDAC2 or HDAC7 by siRNA induce premature senescence. Moreover, the re-expression of HDAC7, but not HDAC2, in pre-senescent fibroblasts allows the extension of their proliferative lifespan. Finally, the effect of HDACs inhibition on *IL-6* and *IL-8* expression is partly dependent on NF-κB activation.

In conclusion, our results demonstrate the role of HDACs in the senescence of dermal fibroblasts (Figure 6). The data strongly suggest that HDACs expression and activity are decreased during replicative senescence and that the inhibition or invalidation of HDACs induces premature senescence. Moreover, the re-expression of *HDAC7* in pre-senescent cells allows the extension of their proliferative lifespan. These results help us to understand the mechanisms associated with ageing and could open new leads in the field of TIS and anti-ageing therapeutic strategies.

## MATERIALS AND METHODS

### Cell culture and treatments

AG04431 HDFs (Coriell Institute) were cultured in BME, supplemented with 10% FBS and 2 mM L-glutamine (Life Technologies) until replicative senescence.

Primary normal human dermal fibroblasts (NHDFs) were isolated from adult abdominal (NHDF1) and young foreskin (NHDF2) samples as previously described [47]. Samples were obtained after plastic surgery (Dr. B. Bienfait, Clinique St-Luc, Bouge Belgium) or circumcision (Dr L. de Visscher, Clinique St-Luc, Bouge, Belgium) following approval by the ethic committee of the Clinique St-Luc (Bouge, Belgium). NHDFs were cultured in BME, supplemented with 10% FBS and 2 mM L-glutamine (Life Technologies) until intermediate replicative lifespan (corresponding to 50% of their proliferative lifespan) and until replicative senescence. All cells were cultured at 37° C in a humidified atmosphere containing 5% CO_2_.

HDFs were studied at early passage (young), i.e. at passages prior the middle of their proliferative lifespan, or when pre-senescent, i.e. at a few passages to entering in replicative senescence, but still proliferating.

Since NHDFs have a longer proliferative lifespan than AG04431, we used an additional passage, called “intermediate”, for these cells corresponding approximately to the middle of their proliferative lifespan.

For SAHA treatment, AG04431 HDFs were treated with 0, 5 or 10 μM SAHA (Selleckchem) during 24, 48 or 72 hr. Fresh medium containing SAHA was renewed every day.

For NF-κB pathway study, AG04431 HDFS were treated with 0 or 10 μM of SAHA during 24, 48 or 72 hr and with 0 or 100 μM of JSH-23 (Sigma-Aldrich) concomitantly during the first 24 hours. Fresh medium containing SAHA was renewed every day. Positive control for NF-ĸB activation was generated by treating HDFs with 20 ng/ml of TNF-α (R&D Systems) during 20 minutes.

### Cell transfection and RNA interference

AG04431 HDFs transfections were performed using siGENOME SMART pool human HDAC2 or HDAC7 (Dharmacon). Non-target siRNA was used as a control for non-specific effect (Dharmacon). Cells were transfected 24 hr under standard culture conditions with 25 nM siRNA using Dharmafect (Dharmacon) transfection reagent according to the manufacturer’s instructions. Cells were cultured under standard culture conditions during 7 days until analysis.

### Lentiviral transduction

Mammalian gene expression lentiviral vectors pLV EF1A EGFP, pLV EF1A hHDAC2, pLV EF1A hHDAC7 were purchased from Vector Builder and were transformed, amplified and purified by GIGA-Viral vectors platform (ULiège). AG04431 HDFs and NHDFs were transduced with a multiplicity of infection (MOI) of 50 in fresh medium containing protamine sulfate (8 μg/ml, MP biochemicals). At 24 hr after the transduction, the medium was replaced with fresh medium. At confluency, cells were subcultured until they reached replicative senescence.

### Senescence-associated beta-galactosidase (SA-βgal) activity

Cells were seeded in 6-well plates (Corning) and the next day, SA-βgal staining was performed as described in [48, 49] during 16 hr. The proportion of SA-βgal positive cells was quantified by counting at least 300 cells per well.

### RNA isolation and real time PCR

Total RNA was isolated using the ReliaPrep cell and tissue miniprep kit (Promega) and reverse-transcribed using GoScript Reverse Transcriptase Kit (Promega) following manufacturer’s instructions. Real-time polymerase chain reaction (PCR) was performed using GoTaq qPCR Master Mix (Promega), primers and the Viia7 Real-Time PCR system (Applied Biosystems). Primers used are referenced in Supplementary Table 1. Relative abundance was determined with the ΔΔCq method [50] normalized to the mRNA abundance of *GAPDH* and expressed relative to the stated control.

### Western blot analysis

Total cell protein lysates were obtained using a buffer containing 40 mM TrisHydroxyMethyl (Tris, Merck Millipore), 150 mM KCl (Merck Millipore), 1 mM EDTA (Merck Millipore), pH 7.5, 1% Triton X-100 (Sigma-Aldrich), supplemented with Complete Protease Inhibitor Cocktail (Roche) and 4% phosphatase inhibitor buffer (25 mM Na_3_VO_4_ (Sigma-Aldrich), 250 mM 4-nitrophenyl phosphate (Sigma-Aldrich), 250 mM β-glycerophosphate (VWR), 125 mM NaF (Merck Millipore)). Protein concentration was determined by Pierce 660 nm Protein Assay (Thermo Scientific). 5 to 10 μg of proteins were separated by SDS-PAGE on 10% SDS-PAGE (NuPAGE, Thermo Fisher) gels and electrotransferred on a polyvinylidene fluoride membrane (Merck Millipore) or on a nitrocellulose membrane (Merck Millipore). Molecular weights were determined with colour pre-stained protein standard (New England Biolabs). Membranes were blocked 1 hr at room temperature in Odyssey blocking buffer (LI-COR) and then overnight at 4° C in Odyssey blocking buffer with 0.1% Tween 20 (Bio-Rad) containing a primary antibody. Membranes were incubated with the secondary antibody for 1 hr in Odyssey blocking buffer with 0.1% Tween 20. Then the membranes were dried 1 hr at 37° C and scanned using Image Studio Lite software V3.1.4 (LI-COR) for quantification. Antibodies used in this study are referenced in Supplementary Table 2.

### Immunofluorescence

Cells were fixed with paraformaldehyde 4% (PFA, Merck Millipore) during 10 min. Cells were then permeabilized with PBS + 1% Triton-X100 (Sigma-Aldrich) during 5 min. Cells were washed in PBS containing 2% Bovine Serum Albumin (BSA, Santa Cruz Biotechnology) 3 times during 10 minutes. Glass coverslips were then incubated in PBS + BSA 2% containing a primary antibody overnight at 4° C. Cells were incubated with PBS + BSA 2% containing a secondary antibody at room temperature in a wet room. Cells were then stained with DAPI (1 ng/ml in PBS, Sigma-Aldrich) for 5 minutes in room temperature. Coverslips were then mounted on superfrost microscope slides using Mowiol (Sigma-Aldrich) and analysed by confocal microscopy (Leica). Antibodies used are referenced in Supplementary Table 2.

### Enzyme-linked immunosorbent analysis (ELISA)

Supernatants were collected after SAHA treatment and kept in -80° C until the test. IL-6 and IL-8 secreted levels were analysed using Human IL-6 Quantikine® Elisa (R&D Systems) and Human IL-8 Quantikine® Elisa (R&D Systems) following manufacturer’s instructions. IL-6 and IL-8 secreted levels were quantified relative to total protein content.

### Statistical analysis

Experiments were performed at least 3 times independently except when noted otherwise. Results are expressed as mean +/- SD (standard deviation). Statistical analyses were performed by using *t*-test, ANOVA I or ANOVA II (GraphPad Prism® Software) (NS: non significant; *: p<0.05; **: p<0.01; ***: p<0.001).

## Supplementary Material

Supplementary Method

Supplementary Figures

Supplementary Tables
