# Supplementary material for: HDAC2 and 7 down-regulation induces senescence in dermal fibroblasts
Source: Aging (Albany NY). 2021 Jul 12;13(14):17978–8005. doi: 10.18632/aging.203304 (PMC8351730; doi:10.18632/aging.203304)
Supplement: Supplementary Method [file aging-13-203304-s001.pdf]

SUPPLEMENTARY METHOD

MTT assay

AG04431 HDFs were treated with 0, 5 or 10 µM SAHA (Selleckchem) during 24, 48 or 72 hours and MTT assay was performed every 24 hours. Cells were incubated with MTT (Sigma-Aldrich) during 2 hours and then lysed in dimethylsulfoxide (DMSO, Carl Roth). The absorbance was read with a spectrometer at 570 nm.
