# Supplementary material for: HDAC2 and 7 down-regulation induces senescence in dermal fibroblasts
Source: Aging (Albany NY). 2021 Jul 12;13(14):17978–8005. doi: 10.18632/aging.203304 (PMC8351730; doi:10.18632/aging.203304)
Supplement: Supplementary Figures [file aging-13-203304-s002.pdf]

SUPPLEMENTARY FIGURES

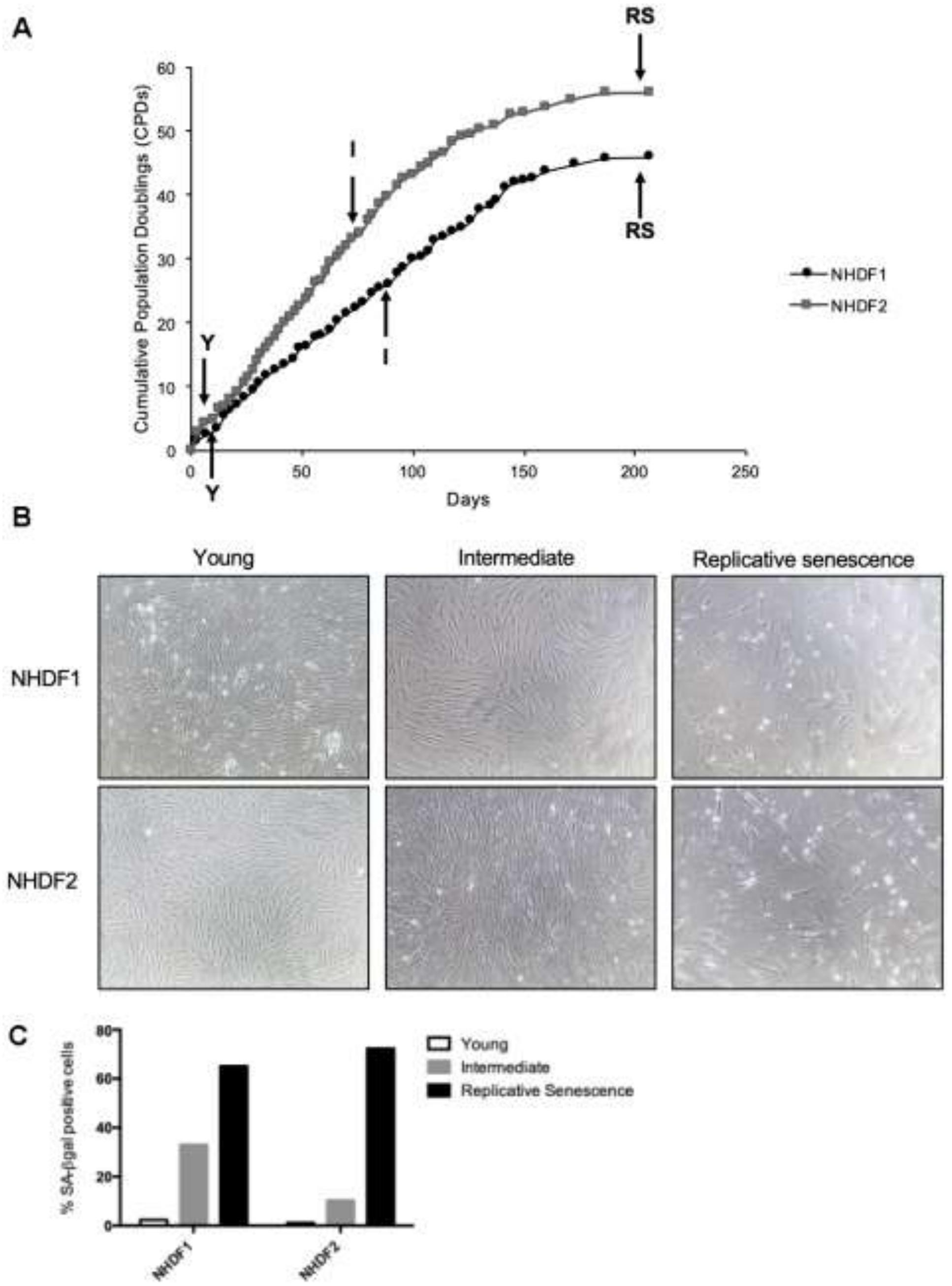

Supplementary Figure 1. Characterization of the phenotype of Normal Human Dermal Fibroblasts (NHDFs). Cells were isolated from two independent donors (NHDF1, 57-years old donor; NHDF2, 3-years old donor) and were passaged in culture until reaching replicative senescence (RS). Analyses were performed at the beginning of the culture (Young, Y), at intermediate passage, namely at the middle of their proliferative lifespan (Intermediate, I) or at the end of the culture (replicative senescence, RS). (**A**) Representative growth curves of the cells with indicated conditions. The passages studied for the experiments are indicated by an arrow. (**B**) Representative bright-field pictures of NHDFs. 100x magnification. (**C**) Percentage of SA-βgal positive cells.

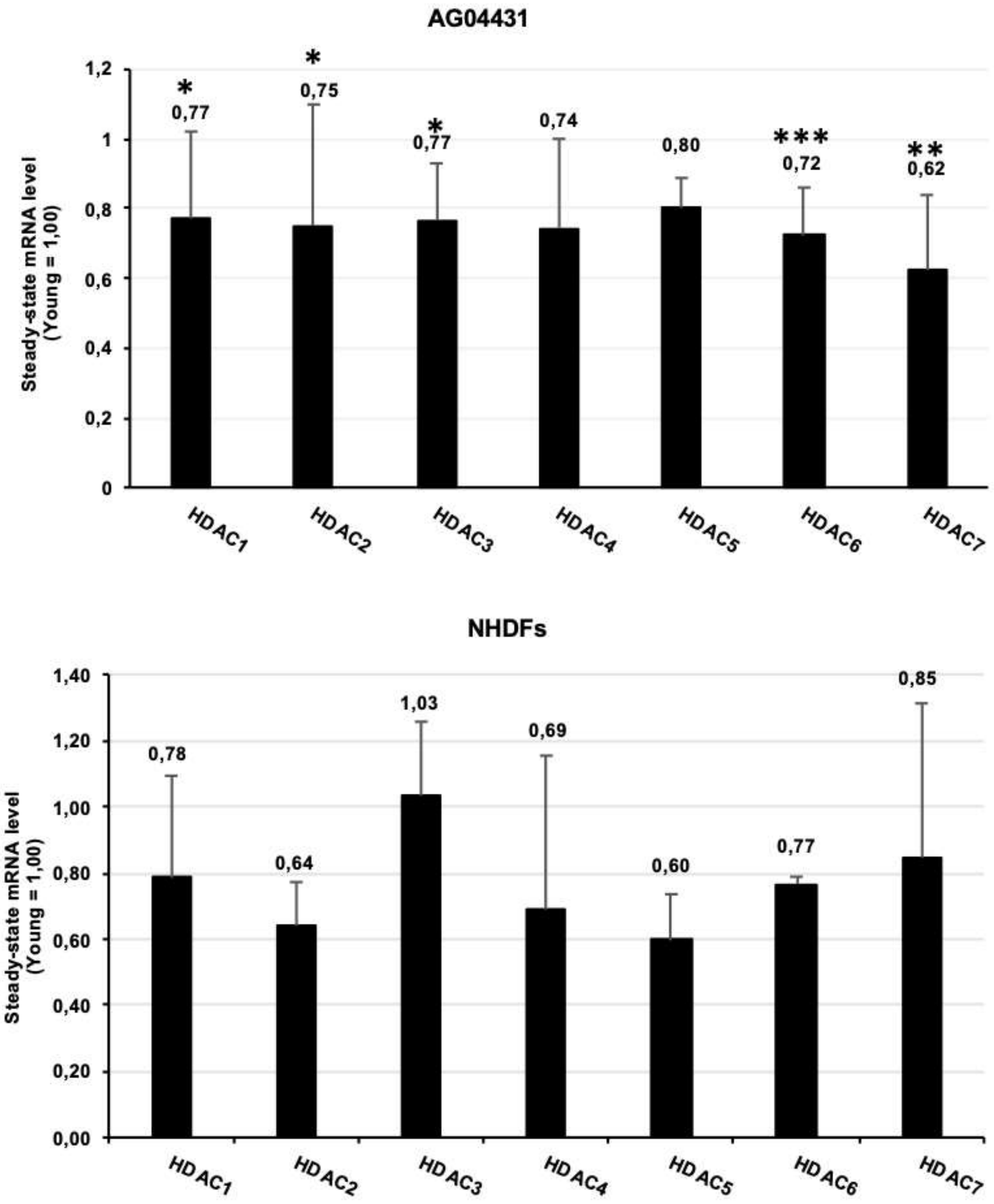

Supplementary Figure 2. Reduced gene expression of *HDACs* in replicative senescence. Steady-state mRNA level of *HDACs 1-7. GAPDH* was used as housekeeping gene. Results are expressed as fold induction in comparison with young cells (N=4 for AG04431, N=2 for NHDFs). Statistical analyses were performed using *t*-test (*: 0,01 < p < 0.05; **: 0.001 < p< 0.01).

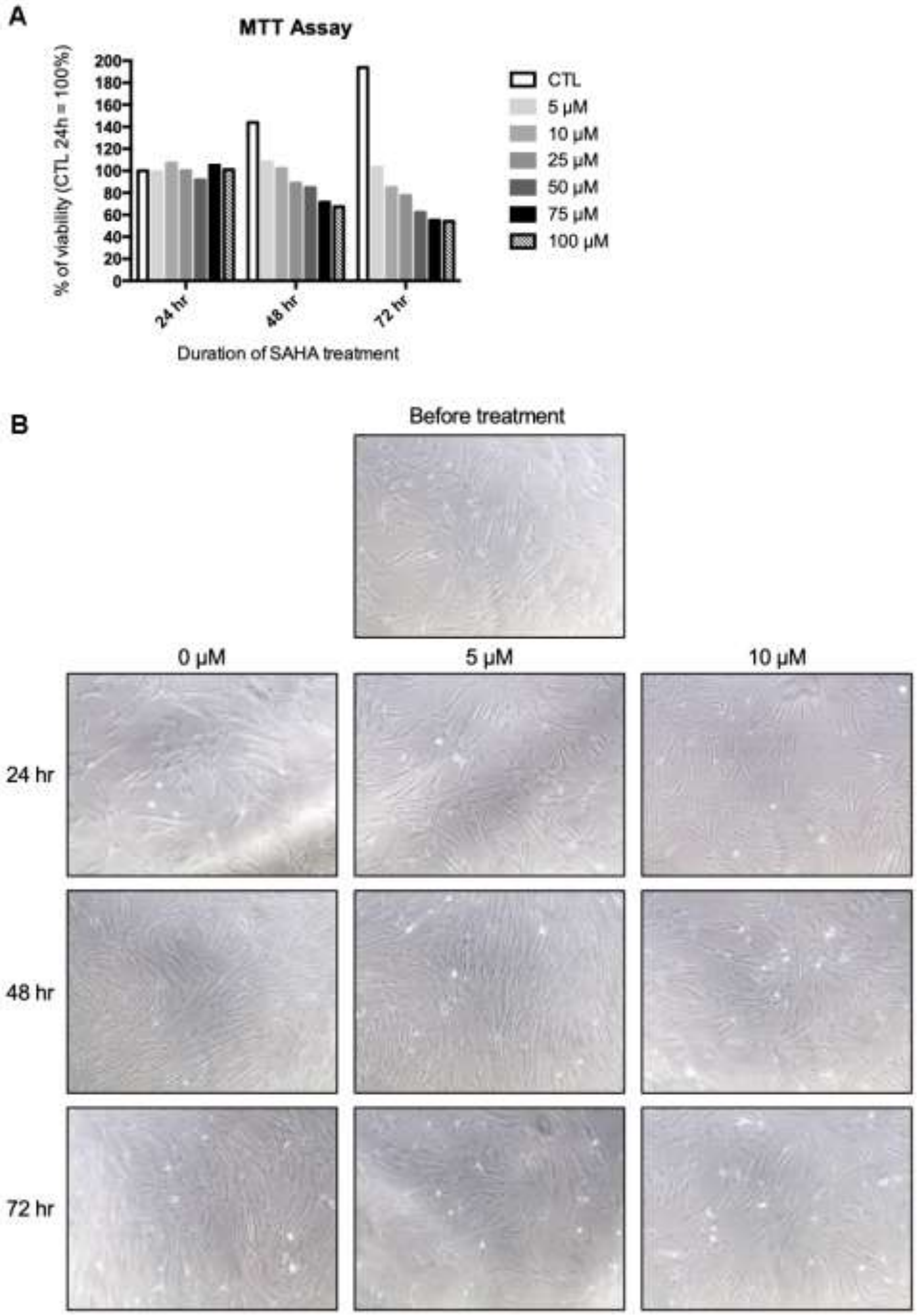

Supplementary Figure 3. Cell viability after SAHA treatment. AG04431 cells at early passage were treated with 0 to 100 µM SAHA during 24, 48 or 72 hr (**A**) 3-(4,5-dimethylthiazol-2-yl)-2,5-diphenyltetrazolium bromide (MTT) assay. Results are expressed relative to the control condition (0 µM SAHA, 24 hr). (**B**) Representative bright-field pictures of AG04431 cells treated with 0, 5 or 10 µM SAHA during 24, 48 or 72 hr. 100x magnification.

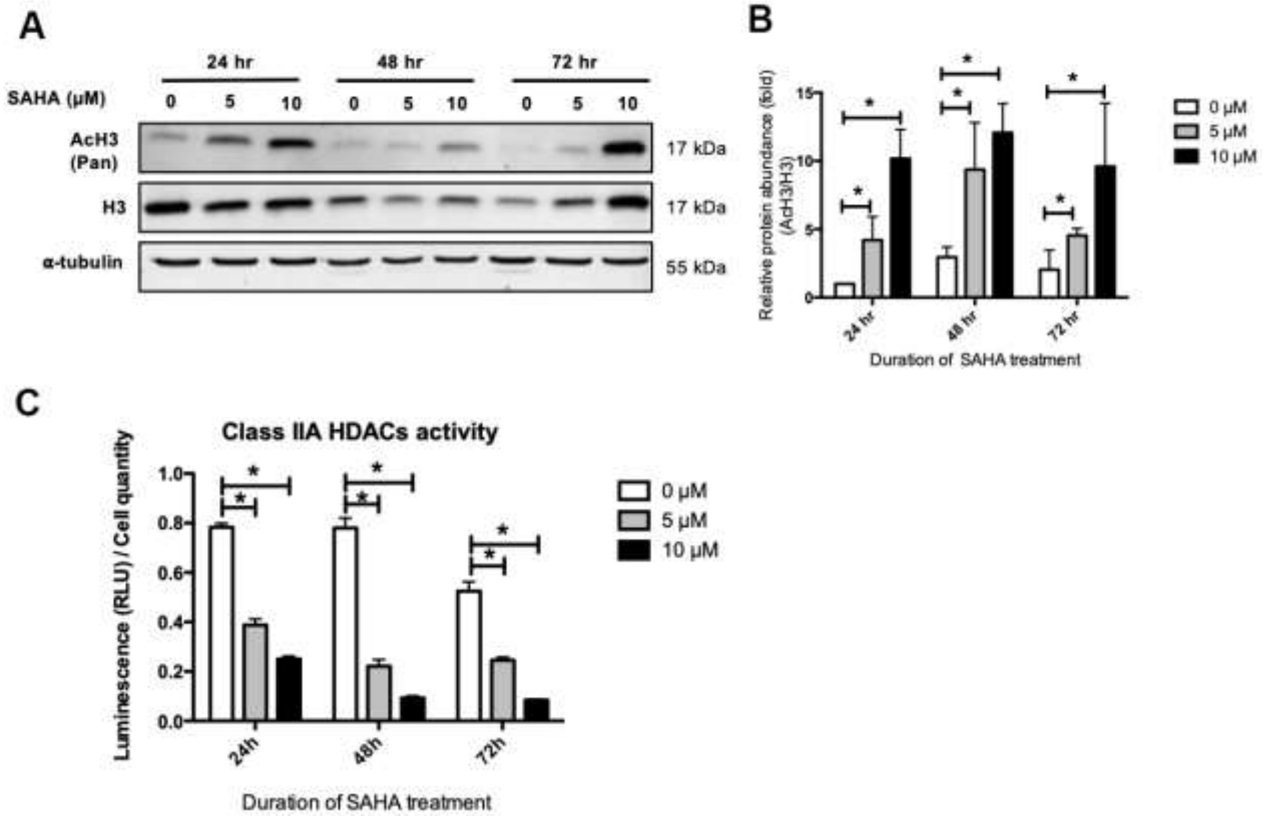

Supplementary Figure 4. Increased Histone H3 acetylation following SAHA treatment of AG04431 cells. Cells at early passage were treated with 0, 5 or 10 µM of SAHA during 24, 48 or 72 hr. (**A**) Representative western blots showing acetylated Histone 3 (AcH3, pan-lysine), with α-tubulin as loading control. (**B**) Quantification of the relative protein abundance of acetylated Histone 3 (pan-lysine) related to histone H3. Signal intensities were quantified and normalised to the abundance of α-tubulin and are expressed relative to the control condition (0 µM SAHA, 24 hr). (**C**) Class IIA HDACs activity assay.

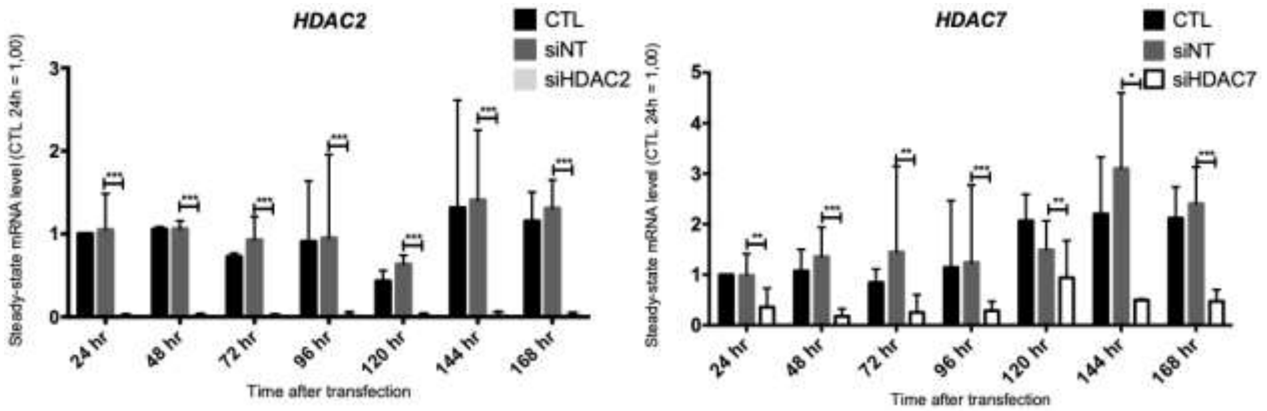

Supplementary Figure 5. *HDAC2* or *HDAC7* knockdown strongly reduces their transcript level. AG04431 cells at early passage were transfected with control siRNA (non target, siNT), HDAC2 siRNA or HDAC7 siRNA during 24 hours. Steady- state mRNA level of *HDAC2* and *HDAC7*. *GAPDH* was used as housekeeping gene. Results are expressed as fold induction in comparison with control fibroblasts (not transfected) at 24 hr. Statistical analyses were performed using ANOVA II (*: 0,01< p < 0.05 ; **: 0.001< p< 0.01; ***: p < 0,001).

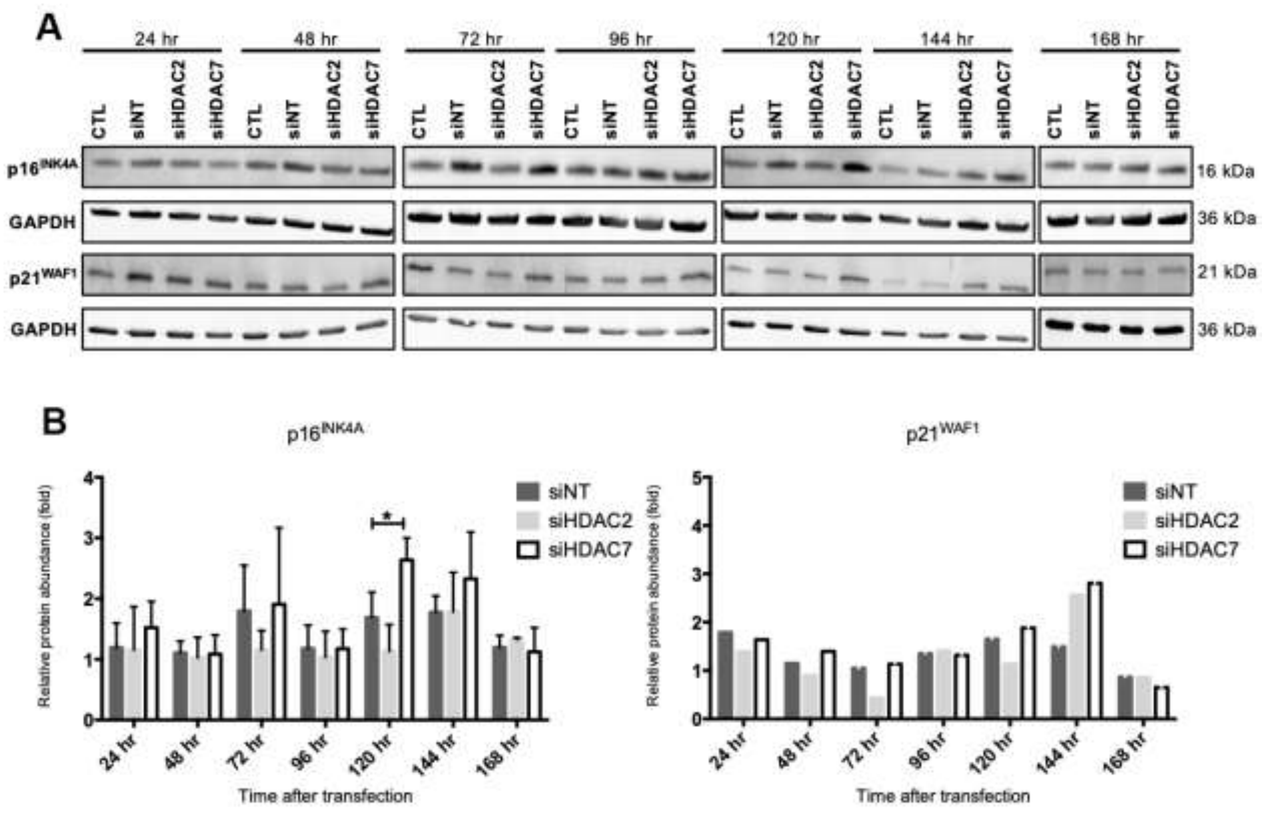

Supplementary Figure 6. *HDAC2* or *HDAC7* knockdown slightly increases protein level of p16^{INK4A} and p21^{WAF1}. AG04431 cells at early passage were transfected with control siRNA (non target, siNT), HDAC2 siRNA or HDAC7 siRNA for 24 hours. (**A**) Representative Western blots showing p16^{INK_4a} and p21^{WAF-1} expression, with GAPDH as loading control (the loading control is the same for p16 as for HDAC2 and HDAC7 (Figure 3A) as it is the same membrane). (**B**) Quantification of the relative protein abundance of p16^{INK4A} (N=3) and p21^{WAF1} (N=2). Signal intensities were quantified and normalized to the abundance of GAPDH and are expressed relative to control fibroblasts at 24 hours. Statistical analyses were performed using ANOVA II (*: 0,01< p < 0.05 ; **: 0.001< p< 0.01; ***: p < 0,001).

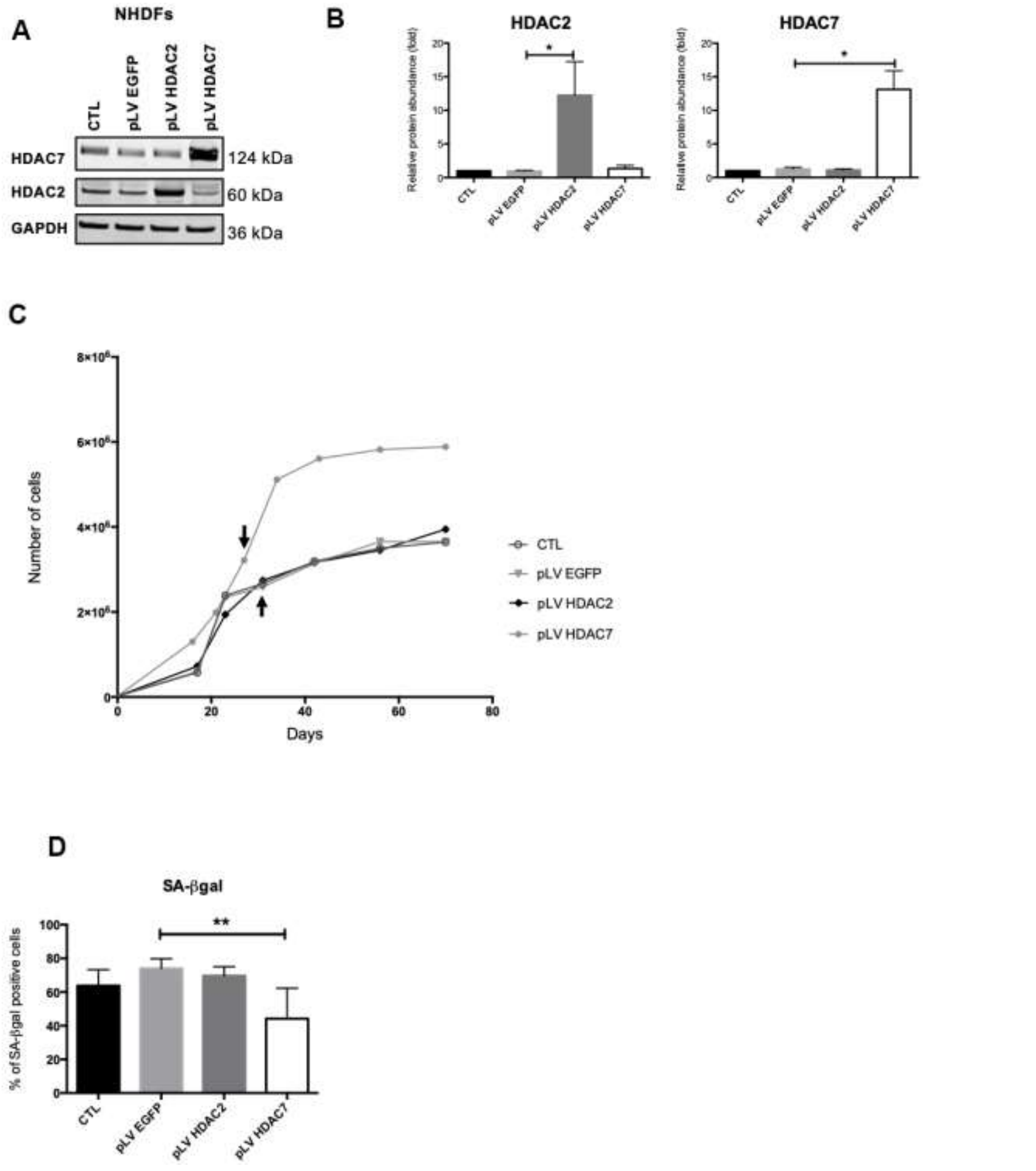

Supplementary Figure 7. HDAC7 but not HDAC2 re-expression allows to resume proliferation and to decrease the proportion of SA-βgal positive cells in pre-senescent cells. Pre-senescent NHDFs, i.e. cells at few passages from the onset of replicative senescence, were transduced with lentiviruses expressing EGFP (pLV EGFP), HDAC2 (pLV HDAC2) or HDAC7 (pLV HDAC7). (**A**) Representative Western blots showing HDAC2 and HDAC7 expression after transduction. GAPDH was used as a loading control. (**B**) Quantification of the relative protein abundance of HDAC2 and HDAC7. Signal intensities were quantified and normalized relative to the abundance of GAPDH and are expressed relative to the control condition (CTL). (**C**) Representative growth curves of the cells with indicated conditions. The passages studied for the experiments are indicated by an arrow. (**D**) Percentage of SA-βgal positive cells.

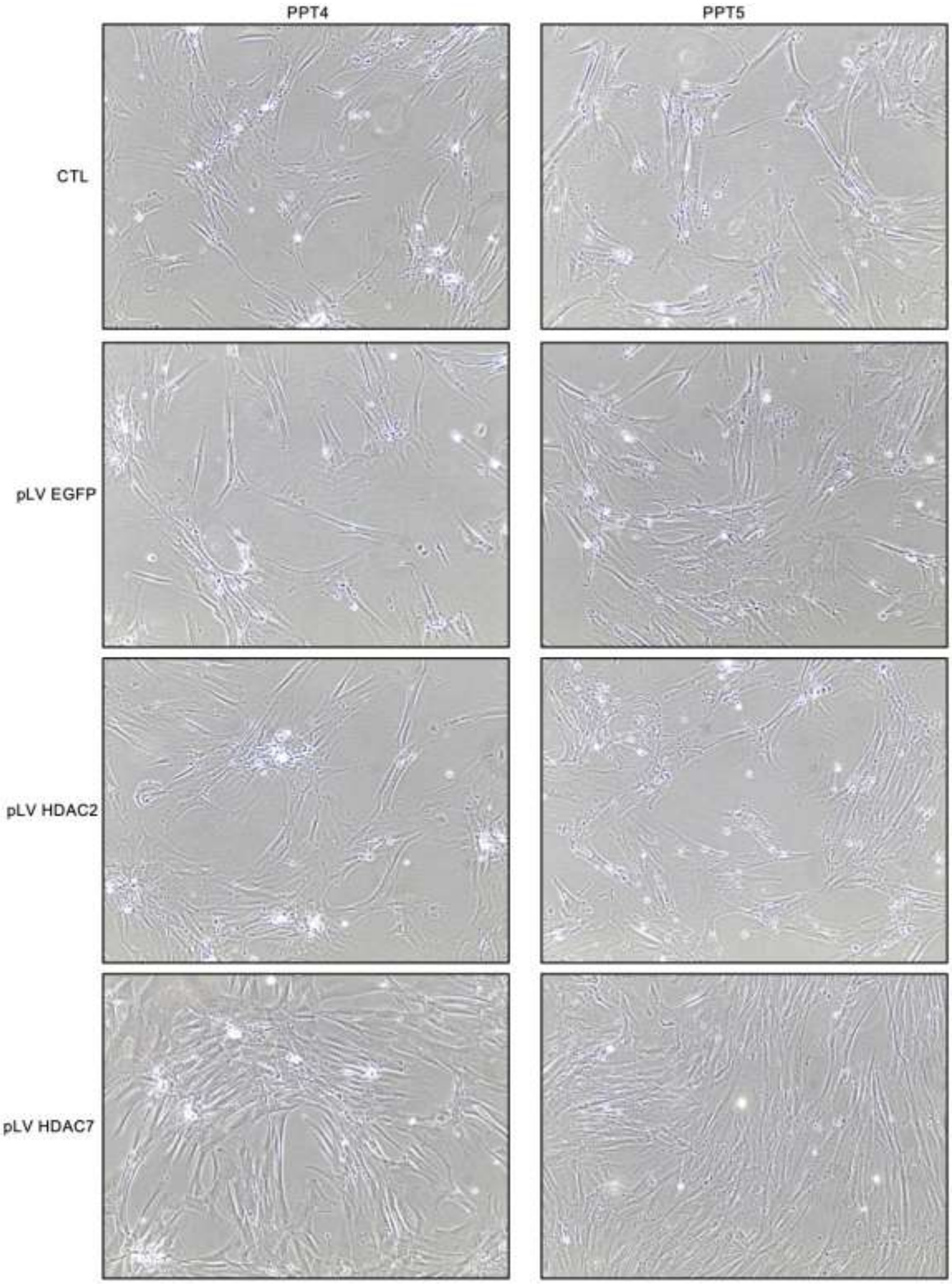

Supplementary Figure 8. HDAC 7 but not HDAC2 re-expression maintains young spindle-shaped morphology in pre-senescent cells. AG04431 cells were transduced with lentiviruses expressing EGFP (pLV EGFP), HDAC2 (pLV HDAC2) or HDAC7 (pLV HDAC7). Representative bright-field pictures at different passages following transduction. 100x magnification. PPT, Passage post-transduction.

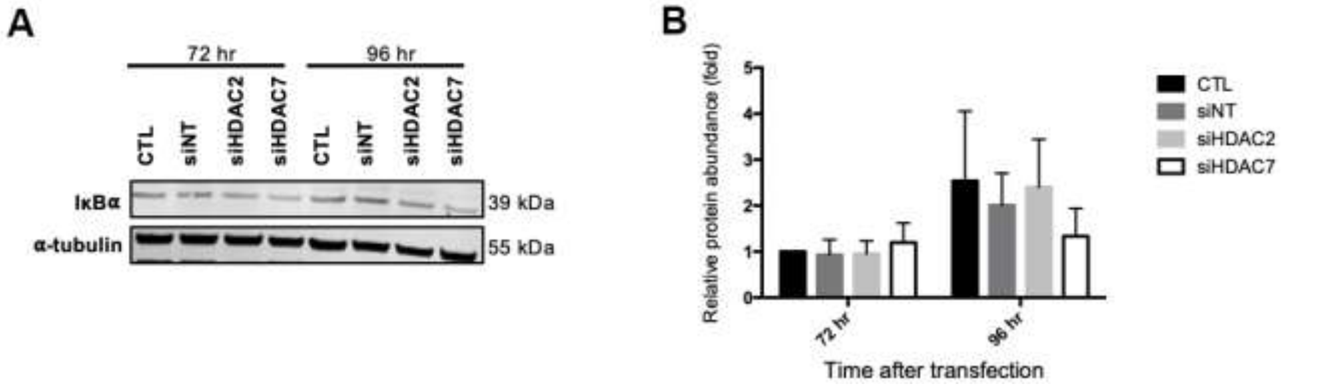

Supplementary Figure 9. IL-6 and IL-8 expression observed after *HDAC2* or *HDAC7* knockdown are partly dependent on NF-κB activation. Cells at early passage were transfected with control siRNA (non target, siNT), HDAC2 siRNA or HDAC7 siRNA during 24 hr. (**A**) Representative Western blots showing IκBa total protein abundance at 72 and 96 hr after siRNA transfection. (**B**) Quantification of the relative protein abundance of IκBa. Signal intensities were quantified and normalized relative to the abundance of α-tubulin and are expressed relative to the control condition (CTL 72h). Statistical analyses were performed using ANOVA II (*: 0,01< p < 0.05; **: 0.001< p< 0.01).
