# Supplementary material for: HDAC2 and 7 down-regulation induces senescence in dermal fibroblasts
Source: Aging (Albany NY). 2021 Jul 12;13(14):17978–8005. doi: 10.18632/aging.203304 (PMC8351730; doi:10.18632/aging.203304)
Supplement: Supplementary Tables [file aging-13-203304-s003.pdf]

SUPPLEMENTARY TABLES

Supplementary Table 1. Primers used for RT-qPCR.

Abbreviation	Genes	Forward primer (5'-3')	Reverse Primer (5'-3')
CLU	Apolipoprotein J	GGA TGA AGG ACC AGT GTG ACA AG	CAG CGA CCT GGA GGG ATT C
GAPDH	Glyceraldehyde-3-Phosphate dehydrogenase	ACC CAC TCC TCC ACC TTT GAC	GTC CAC CAC CCT GTT GCT GTA
HDAC 1	Histone deacetylase 1	CTA TCA AAG GAC ACG CCA AGT G	ACC GGG CAA CGT TAC GAA T
HDAC 2	Histone deacetylase 2	CAT GGT GAT GGT GTT GAA GAA G	TCA TTG GAA AAT TGA CAG CAT AGT
HDAC 3	Histone deacetylase 3	TTG AGT TCT GCT CGC GTT ACA	CCC AGT TAA TGG CAA TAT CAC AGA T
HDAC 4	Histone deacetylase 4	GTA AGA AAC TTC TAG GCT CGC TC	ACC TCG TTC CAT ATG GTG TCA
HDAC 5	Histone deacetylase 5	TTG GAG ACG TGG AGT ACC TTA CAG	GAC TAG GAC CAC ATC AGG TGA GAA C
HDAC 6	Histone deacetylase 6	TGG CTA TTG CAT GTT CAA CCA	GTC GAA GGT GAA CTG TGT TCC T
HDAC 7	Histone deacetylase 7	CTG GCA CAG CGG ATG TTT G	CTG CAT TGG AGG AAT GAA GCT
IL-6	Interleukin-6	TCC AGG AGC CCA GCT ATG AA	CCC AGG GAG AAG GCA ACT G
IL-8	Interleukine-8	CTG GCC GTG GCT CTC TTG	GGG TGG AAA GGT TTG GAG TAT G
MMP-1	Metalloproteinase-1	CAT GCG CAC AAA TCC CTT CTA	GAA CAG CCC AGT ACT TAT TCC CTT T
MMP-3	Metalloproteinase-3	TTT GGC CCA TGC CTA TGC	CCA GGG AGT GGC CAA TTT C
$p21^{WAF1}$	Cyclin-dependent Kinase Inhibitor 1	CTG GAG ACT CTC AGG GTC GAA	CCA GGA CTG CAG GCT TCC T
$p16^{INK4a}$	Cyclin-dependent Kinase Inhibitor 2a	GCC CAA CGC ACC GAA TAG T	CGC TGC CCA TCA TCA TGA C
LMNB1	Lamin B1	ACTGGCGAAGATGTGAAGGTTAT	CCCTGCTGGTGGAAAAGTTC
IL-1β	Interleukine-1β	GCCCTAAACAGATGAAGTGCTC	GAGATTCGTAGCTGGATGCC
CXCL1	Chemokine (C-X-C) motif 1 (Gro-α)	CGAAAAGATGCTGAACAGTGAC	ACATTAGGCACAATCCAGGTG

Supplementary Table 2. Antibodies used for western blotting and immunofluorescence.

Antibody	Firm	Isotype	Source	Dilution	Code
HDAC 1	Cell Signaling Technology	Monoclonal	Mouse	1/1000	1OE2 #5356
HDAC 2	Cell Signaling Technology	Monoclonal	Mouse	1/1000	3F3 #2540
HDAC 3	Cell Signaling Technology	Monoclonal	Mouse	1/1000	7G6C5 #3949
HDAC 4	Cell Signaling Technology	Monoclonal	Rabbit	1/1000	D15C3 #7628
HDAC 5	Cell Signaling Technology	Monoclonal	Rabbit	1/500	D17V #2082
HDAC 6	Cell Signaling Technology	Monoclonal	Rabbit	1/1000	D2E5 #7558
HDAC 7	Cell Signaling Technology	Monoclonal	Rabbit	1/1000	D4E1L#33418
GAPDH	Abcam	Monoclonal	Rabbit	1/10 000	EPR6256
$p16^{INK4a}$	Abcam	Monoclonal	Rabbit	1/2000	#108349
$p21^{WAF1}$	Cell Signaling Technology	Monoclonal	Mouse	1/1000	DCS60 #2946
Histone H3 (Pan-Acetyl)	Active Motif	Polyclonal	Rabbit	1/1000	39139
Acetylated lysine	Cell Signaling Technology	Polyclonal	Rabbit	1/1000	#9441
Histone H3	Cell Signaling Technology	Polyclonal	Rabbit	1/1000	#9715
IκBα	Santa Cruz Technology	Monoclonal	Rabbit	1/500	#sc-1643
Alpha-tubulin	Sigma	Monoclonal	Mouse	1/20 000	T5168
IRDye800CW Goat anti-mouse IgG	LI-COR	Polyclonal	Goat	1/10 000	#926-32210
IRDye800CW Goat anti-rabbit IgG	LI-COR	Polyclonal	Goat	1/10 000	#926-32211
HDAC7	Cell Signaling Technology	Monoclonal	Rabbit	1/100	D4E1L#33418
HDAC2	Cell Signaling Technology	Monoclonal	Mouse	1/400	3F3 #2540
Ki-67	Cell Signaling Technology	Monoclonal	Mouse	1/800	#9449
p65	Cell Signaling Technology	Monoclonal	Rabbit	1/400	#8242
Anti-mouse Alexa 488nm	Molecular Probes	Polyclonal	Goat	1/1000	#A-11001
Anti-rabbit Alexa 488nm	Molecular Probes	Polyclonal	Goat	1/1000	#A-11008
